# Investigation of irradiated volume in linac-based brain hypo-fractionated stereotactic radiotherapy

**DOI:** 10.1186/s13014-017-0853-5

**Published:** 2017-07-14

**Authors:** Mark Ruschin, Arjun Sahgal, Hany Soliman, Sten Myrehaug, May Tsao, Collins Yeboah, Arman Sarfehnia, Brige Chugh, Alex Kiss, Young Lee

**Affiliations:** 1 Department of Radiation Oncology, Sunnybrook Health Sciences Centre, Toronto, ON Canada; 2 Department of Radiation Oncology, University of Toronto, Toronto, ON Canada; 3 Research Design and Biostatistics, Sunnybrook Health Sciences Centre, Toronto, ON Canada

**Keywords:** Brain, Hypofractionated, Radiotherapy, Radiosurgery, Metastasis, Irradiated volume

## Abstract

**Background:**

Emerging techniques such as brain hypo-fractionated radiotherapy (HF-RT) involve complex cases with limited guidelines for plan quality and normal tissue tolerances. The purpose of the present study was to statistically parameterize irradiated volume independently of dose prescription, or margin to determine what spread in achievable irradiated volume one may expect for a given case.

**Methods:**

We defined EXT as the total tissue within the external contour of the patient (including the target) and we defined BMP as the contour of the brain minus PTV. Irradiated volumes of EXT and BMP at specific doses (i.e. 50, 60%, etc., of the prescribed dose) were extracted from 135 single-target HF-RT clinical cases, each planned with a single-arc, homogeneous (SAHO) approach in which target maximum dose (Dmax) was constrained to <130% of the prescribed dose. Irradiated volumes were subsequently measured for cases involving 2 targets (*N* = 29), 3 targets (*N* = 7) and >3 targets (*N* = 10) to investigate the effect of target number. We also examined the effect of shape complexity. A series of best fit curves with confidence and prediction intervals were generated for irradiated volume versus total target volume and the resulting model was subsequently validated on a subsequent set of 23 consecutive prospective cases not originally used in curve-fitting. A subset of 30 HF-RT cases were re-planned with a well-published four-arc, heterogeneous (FAHE) radiosurgery planning approach (Dmax could exceed 130%) to demonstrate how technique affects irradiated volume.

**Results:**

For SAHO, strong correlation (R^2^ > 0.98) was found for predicting irradiated volumes. For a given total target volume, irradiated-volume increased by a range of 1.4–2.9× for >3 versus single-targets depending on isodose level. Shape complexity had minor impact on irradiated volume. There was no statistical difference in irradiated volumes between validation and input data (*p* > 0.2). The FAHE-generated irradiated volumes yielded curves and prediction and confidence bands that agreed well with published data indicating that the proposed approach is feasible for cross-institutional comparisons.

**Conclusions:**

A description of irradiated volume for linac-based HF-RT is proposed based on population data. We have demonstrated that the proposed approach is feasible for inter and intra-institutional comparisons.

## Background

Evidence continues to support the safety and efficacy of stereotactic radiosurgery (SRS) [1, 2] and emerging techniques such as hypo-fractionated radiotherapy (HF-RT) [3–5]. For SRS, recent clinical evidence supports the safety in treating up to ten targets in a single fraction [1]. For HF-RT, radiation-induced harm to normal tissue, in particular for large or recurrent disease, is mitigated whilst delivering a clinically effective dose to the target [4–7]. In conjunction with modern treatment planning systems, linear accelerators equipped with high-definition multileaf collimators (MLCs), image-guidance and robotic couches, both SRS and HF-RT are increasing in utility.

Cases are becoming more challenging with centres treating large number of targets, large volumes, recurrent disease, complex shapes, etc. The well-known safety limit of V12 < 10 cm^3^ for SRS [8–10] may not necessarily apply or be feasible for multiple targets, re-treatment scenarios, or HF-RT. The focus in the literature has deservedly been on investigating what technique offers the best rapid dose fall-off: the more rapid the better [11–13]. However, every paper reports their endpoints in a different way, making inter-institutional comparisons challenging if not impossible. Other approaches involve developing predictive models of dose fall-off [14–16]. The work by Shiraishi et al. develops and validates a knowledge-based approach of an accurate model, however the model itself is hidden to the reader [14]. The approach by Bohoudi et al., on the other hand is tailored specifically towards V12 [16]. Furthermore, there are limited published data specific to HF-RT. In one paper by Ruschin et al., irradiated volume is reported as a function of target volume but only for specific dose-fractionation schemes and only for Brain Minus CTV volume, which can be different amongst institutions who apply different PTV margins than those used [17].

In the present study, we propose a framework that all clinics can easily access to evaluate irradiated volume for arbitrary isodose levels; for both untreated brain (Brain Minus PTV) and total tissue; for varying target numbers and shapes. Although irradiated volume depends on many factors such as machine characteristics and technique, we sought to provide a framework through which our institution and others can readily compare population data to each other. The framework can also be applied to prospective individual cases, in which some guidance as to “what is achievable” for that case could be of assistance. Internally, such a framework could provide the basis for treatment plan quality assurance (QA), especially for challenging or off-protocol cases. Additionally, investigating population trends over time can lead to more consistent and higher quality treatment plans. The intention is not to precisely predict irradiated volume for all institutions and techniques, but rather to provide the framework through which irradiate volume can be compared across institutions.

## Materials and methods

### Irradiated volume definition

As shown by previous publications, the volume of tissue receiving specific isodose is proportional to the total target volume raised to an exponent [15, 17]. The primary framework is thus to define irradiated volume for a series of HF-RT patients as [15, 17]:

1 $$ {V}_{primary}(P)= a(P){\left( P TV\right)}^{b(P)} $$

where V_primary_(P) is volume of the external contour (EXT, entire volume inside the patient surface, including skull, skin, etc.) or Brain-Minus-PTV (BMP, the brain contour minus all target volumes) receiving dose P (percentage of prescription), PTV is the total target volume, and a(P) and b(P) are empirically determined fit-parameters for single, regularly-shaped targets. We use PTV rather than GTV in the present manuscript since PTV margins may vary between centres, which could cause differences in reported Brain-Minus-GTV values for a given target size. Although BMP may have more clinical relevance in terms of correlating with brain toxicity outcomes, EXT is a quantity the all treatment planning systems are capable of producing and in some cases this is the only quantity readily available as it does not depend on a contour of the brain being present. Note that by definition, the BMP volume will always be less than the corresponding EXT volume for the same target volume, since the target volume is included in EXT but subtracted to produce BMP. The framework assumes that the target is covered by at least 95% and up to 100% of the prescribed dose, which is common practice in intra-cranial radiotherapy and radiosurgery. The framework also includes the 95% prediction and confidence intervals (PI95 and CI95), which can be determined using a linear regression model. Note that the PI95 is by definition always larger than the CI95 as it is used to forecast individual measurements, rather than compare populations. The PI95 (rather than the CI95) is taken to be the error term of the fit, as the general application of the framework is to forecast future plans, however where appropriate the CI95 is also used to compare to populations together.

### Effect of target number and shape

In order to improve curve-fitting accuracy, it may be useful to stratify cases according to specific situations. For example, target shape and number have been discussed in the literature as potentially affecting irradiated volume [18]. Rather than present Eq. (1) for every possible situation, which could get confusing, our proposed framework is for a centre to first build a model based on single, regularly-shaped targets and then scale that primary model by multiplicative factors. In the present manuscript we examined the effect of number of targets and shape complexity via adjustment factors F_N_ and R as follows.

### Adjustment factor for number of targets – F_N_

To account for number of targets, we define an adjustment factor – F_N_(P) – as follows:

2 $$ {F}_N(P)\stackrel{\scriptscriptstyle\mathrm{def}}{=}\frac{a\_ N(P){\left( P TV\right)}^{b\_ N(P)}}{a\_1(P){\left( P TV\right)}^{b\_1(P)}}\cong \frac{a\_ N(P)}{a\_1(P)} $$

where N = number of targets (2, 3, or >3), a_1(P), and b_1(P) are the fit parameters for single-targets. Assuming that multiple targets cause a perturbation to the model and in order to reduce the fit to a single-parameter, we intentionally set b_N(P) equal to b_1(P). This assumption was tested in a pilot study by comparing a 2-fit operation to a 1-fit operation and we found the overall relative agreement to be within 10% for total target volumes above 20 cm^3^. We can thus express irradiated volume for multiple targets the irradiated volume for single-targets times a scalar as follows:

3 $$ V\_ N(P)={F}_N(P)\left[{a}_{1(P)}{\left( P TV\right)}^{b1(P)}\right]={F}_N(P)\times {V}_{primary}(P) $$

where V_primary_(P) is given by Eq. 1. Note: F_N_(P) is always > = 1.

### Adjustment factor for shape regularity–R

To account for shape complexity we define a “regularity index” (RI) that relates how spherical a target is: 1.0 being a perfect sphere and values <1.0 becoming less spherical [17, 18]. Mathematically, we have defined RI as:

4 $$ RI=\frac{2\times {V}_{S, T}}{V_T+{V}_S}=\frac{V_{S, T}}{V_T} $$

where V_T_ is the target volume (PTV in the present study), V_S,T_ is the volume of overlap between the target volume and an equivalent-volume sphere positioned at the centre of gravity of the target volume, and V_S_ is the sphere volume, which by definition is equal to V_T_. Based on preliminary investigations of irradiated volumes, we classified highly irregular shapes as RI < 0.6. Following a similar approach for number of targets, we define an adjustment factor for shape regularity as follows:

5 $$ R(P)=\frac{a_{RI<0.6}(P)}{a_{RI>0.6}(P)} $$

where the a_RI>0.6_(P) is the fit parameter for regularly-shaped targets. We can then express irradiated volume for complex shapes as irradiated volume of regular shapes times a scalar:

6 $$ {V}_{RI<0.6}(P)= R(P)\times {V}_{primary}(P) $$

where V_primary_(P) is given by Eq. 1. Note: R(P) > =1.

### Combined framework for irradiated volume

For a given case involving N targets, of shape complexity RI, planned with a given technique, the combined framework defines the irradiated volume receiving any isodose line P as:

7 $$ V(P)= R(P)\times {F}_N(P)\times {V}_{primary}(P) $$

where the factors R, and F_N_ are used to bring irradiated volume around a single, simple-shaped target to the appropriate shape complexity and number of targets (N) respectively. An error term – δ(P) – is the PI95 around the model. Factors R, F_N_ and δ can thus be characterized as functions of P in order that EXT or BMP receiving any isodose line can be interpolated.

### Framework testing

The framework was applied and tested to two models as follows.

1. **Single-Arc, Homogeneous (SAHO) HF-RT**: 181 consecutive HF-RT cases between 2013 and 2014 were retrospectively accessed under our institutional Review Ethics Board (REB) approval as follows: 1 target (*N* = 135), 2 targets (*N* = 29), 3 targets (*N* = 7), and >3 targets (*N* = 10). Target volumes ranged from 2.3 to 84.6 cm^3^ with a median of 14.1 cm^3^. All HF-RT cases were treated on a Synergy “S” linac (Elekta AB, Stockholm, Sweden) equipped with a 4 mm leaf width MLC at isocentre [19]. Treatment planning was performed using the Pinnacle^3^ treatment planning system (TPS) v9.0 or 9.2 (Philips Healthcare, Andover, USA) with SmartArc optimization for VMAT and using a dose calculation grid of 2 mm. The gross tumor volume (GTV) in the case of intact metastases, and clinical target volume (CTV) in the case of surgical cavities, were contoured on volumetric T1 post-gadolinium MRI fused to the treatment planning CT, which is acquired at 1 mm slice spacing. For intact metastases, no additional expansion to the GTV was made for microscopic spread, therefore CTV = GTV. A PTV margin of 2 mm was applied around each CTV. The target objective was to cover greater than 98% of the PTV (V100 > 98%) with the prescribed dose. The maximum dose in the target was limited to <130% of prescribed dose, and was typically <120%, therefore referred to as being relatively “homogeneous”. A single full arc was used for each case. The prescription dose ranged from 20 to 35 Gy in five fractions. All parameters – F_N_(P), R(P), a(P), b(P) and δ(P) were determined for *P* = 0.5 to *P* = 0.8 for both EXT and BMP based on curve fitting.
2. **Four-Arc, Heterogenous (FAHE)**: A subset of 30 randomly-selected cases was re-planned using the published four-arc technique of Clark et al. and later improved upon by Thomas et al. [12, 20]. In this approach, the same target objective (V100 > 98%) was used, but there were no explicit constraints set for the maximum target dose, which resulted in relatively more “heterogeneous” dose distributions (mean Dmax = 140%, range: 127–158% of the prescribed dose) compared to our institutional protocol, but consistent with the published literature. This technique is commonly used for single-fraction stereotactic radiosurgery (SRS), but we recognized that institutions may translate the same technique over to the larger-target HF-RT cases, which motivated applying our framework to this technique.

### Validation and feasibility

Our clinical SAHO model was validated against 23 consecutive prospective cases from our institution receiving HF-RT that were not part of the modeling process. The 23 validation cases consisted of single-target (*N* = 16), 2-target (*N* = 3), 3-target (*N* = 3) and >3 (*N* = 1) cases, as well as five cases within the single-target group that had an IR < 0.6. The mean total target volume in the validation set was 25.0 cm^3^ (range: 2.5 cm^3^–58.6 cm^3^). For all cases, we used Eq. 7 to determine V_primary_ by dividing the measured V(P) by the appropriate values of F_N_(P) and R(P). We defined a successful prediction to be a given data point falling within PI95 of our model. Additionally, we investigated whether fits made to the validation data fell within the CI95 of our model, which would indicate that the ensemble of validation data and model were statistically equivalent.

As published SRS data tends to follow the FAHE planning approach, we also used our framework to demonstrate that we can feasibly compare irradiated volumes against five independent publications [11, 12, 16, 18, 21]. It is important to note that in Thomas et al. and Bohoudi et al., the reported V12Gy is equivalent to EXT-67 or BMP67 in our series, since 12Gy is 67% of 18Gy, which was the prescribed dose used in those two studies. This further demonstrates the flexibility of our framework to apply to any isodose level irrespective of prescribed dose.

## Results

### SAHO

**Fig. 1:**
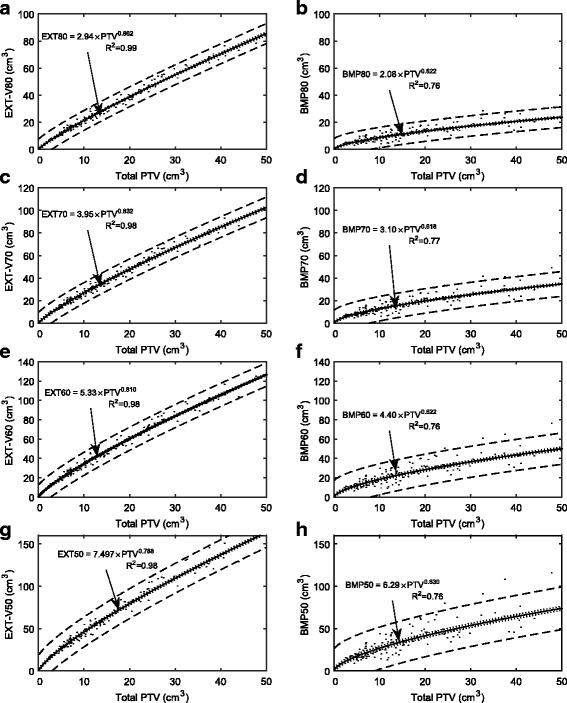
Plots irradiated volume versus target volume using SAHO for single-target data. Left column (**a**, **c**, **e**, and **g**) are plots for the External (EXT) contour. Right column (**b**, **d**, **f**, **h**) are plots for the Brain-Minus-PTV (BMP) contour. Top down to bottom row: shows model for 80%, 70%, 60% and50% of the prescription dose. Each *dot* represents one patient datum point. The best-curve equations according to Eq. (1) (for single-target data) are shown on the graphs. The *dashed* and *dotted lines* indicated the 95% prediction and confidence bands, respectively

The median (inner 95th percentile range) of RTOG conformity index [22], Paddick conformity index [23], and homogeneity index for the 135 plans used in the SAHO model were 1.17 (1.05–1.35), 0.83 (0.73–0.91), and 1.16 (1.08–1.23), respectively. The SAHO model is shown in Fig. 1 as EXT and BMP volumes (i.e. V_primary_(P) as per Eq. 1) receiving doses from 50 to 80% in 10% increments versus target volume. The best-fit curves shown in Fig. 1 were used to obtain coefficients a(P) and b(P) as per Eq. 1. Spearman’s correlation coefficients (R^2^) were larger than 0.98 for the EXT volumes and in the range of 0.76–0.77 for the BMP volumes, indicating a greater degree of spread in the data for BMP fits. The dashed lines indicated the upper and lower bounds of the PI95 while the dotted lines indicated the CI95.

### Number of targets shape regularity and application of FAHE

**Fig. 2:**
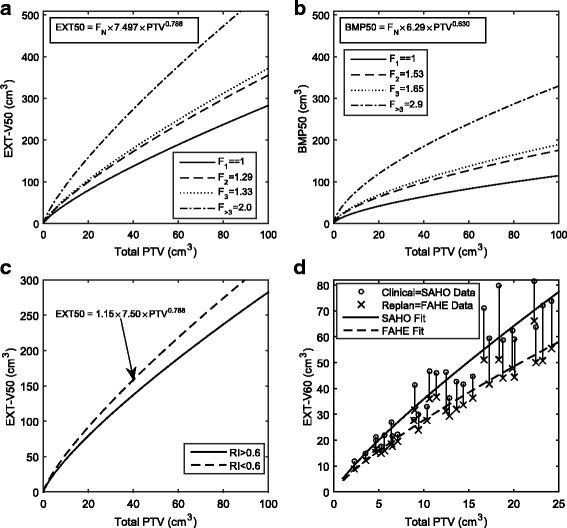
Investigation of number of targets, shape regularity and treatment technique. **a** and **b** are EXT-50 and BMP-50, respectively, versus total PTV for plans with 1 target (primary model) compared to plans with 2, 3, and >3 targets for SAHO. The first term in the equations is the factor F_N_ by which the primary SAHO model is multiplied by to achieve the indicated curve. Individual data points are not shown for ease of interpretation. **c** EXT-50 versus PTV for plans with RI > 0.6 compared to plans with target RI < 0.6. **d** EXT-60 vs Total PTV for a subset of clinical plans re-planned using FAHE. The *arrows* point from the original SAHO plan to the re-plan FAHE plan

The derivation of F_N_ and R is illustrated at the 50% isodose level (*P* = 0.5) in Fig. 2, parts (a) through (c). Note that the equations shown are those from Fig. 1, plus an additional factor that corresponds to the F_N_ or R through curve-fitting and application of Eqs. (2) and (5), respectively.

As shown in Fig. 2d at the 60% isodose level, our FAHE model generally resulted in lower irradiated volumes for any given target volume than our clinical SAHO model. Although not shown in Fig. 2d, FAHE treatment plans consisted of tighter MLC margins around the targets and, as a result of allowing for greater hotspots, greater Dmax relative to the prescribed dose (mean Dmax = 140%, range: 127–158% of the prescribed dose).

**Fig. 3:**
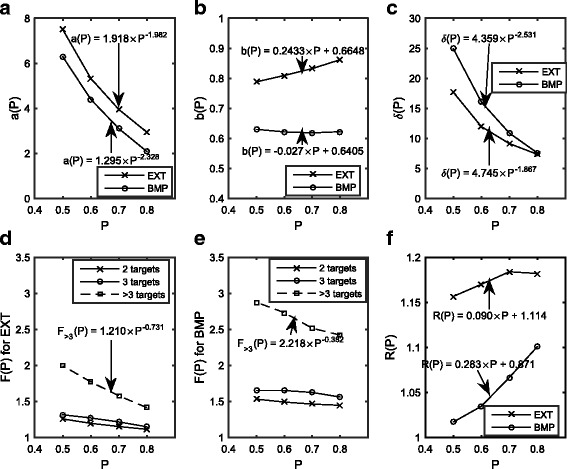
Curve fitting parameters for parameterization of irradiated volume based on the SAHO technique. **a** and **b** show fit parameters **a** and **b** respectively for the primary model as in Eq. (1) and Fig. 1. **c** is the error (δ) equal to the 95% prediction interval. **d** and **e** show the factor F_N_(P) for EXT and BMP respectively. **f** shows the factor R(P)

Putting it all together, each term encompassed by Eqs. 1, 2 and 5, as well as the PI95 (termed δ) are plotted in Fig. 3 as a function of P. As seen in Fig. 3(a) and (b), a(P) lowers for increasing values of P, while the power term b(P) remains relatively flat or modestly increases for BMP and EXT respectively. The derived values of F_N_, R as per Eqs. (2) and (5) are plotted as a function of P in Fig. 3d through f. As seen in Fig. 3(d) through (f), the correction for number of targets (N) in this series is larger than for shape irregularity, with F_N_ approaching a factor of 2 or 3 for *N* > 3 targets in EXT or BMP respectively, while R has a plateau around 1.17. The PI95 (or δ) is higher for BMP than for EXT at all values of P, as shown Fig. 3c, which can also be appreciated by the span of the dashed lines in Fig. 1.

### Validation and feasibility

#### SAHO

**Fig. 4:**
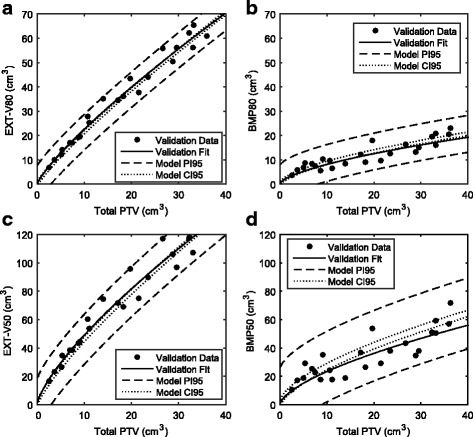
Validation of the irradiated volume framework using SAHO against prospective data at our institution. Parts (**a**) and (**b**) are showing the irradiated volumes receiving 80% of the prescription for EXT and BMP, respectively. Parts (**c**) and (**d**) are showing the irradiated volumes receiving 50% of the prescription for EXT and BMP, respectively. The *dashed lines* are the upper and lower bounds of the 95% prediction interval (PI95) for the primary model. The *dotted lines* are the upper and lower bounds of the 95% confidence interval (CI95) for the SAHO model. EXT = External contour. BMP = BrainMinusPTV

As shown in Fig. 4, at both the 80 and 50% isodose level, all of the data points were encompassed by the model’s PI95 (dashed lines). Furthermore, best fit lines to the validation data for EXT-80 and EXT-50 were observed to fall within the model’s CI95 (dotted lines), indicating that the model and validation data were not statistically different (*p* > 0.2). For BMP-80 and BMP-50, the best fit lines to the validation data fell below the CI95 but were still determined to be statistically equivalent when applying a Student T-test analysis (*p* > 0.2).

#### FAHE

**Fig. 5:**
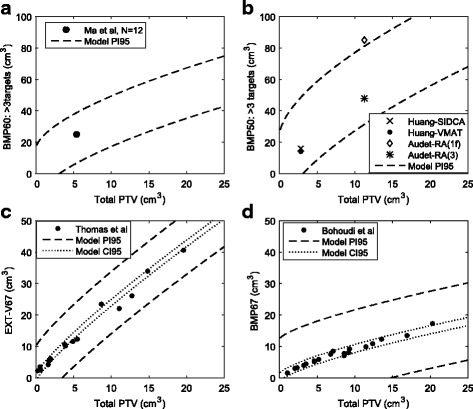
Feasibility of applying irradiated volume framework to compare against published data. Each part corresponds to a particular metric found in the literature as follows: (**a**) is BMP60 for >3 targets; (**b**) is BMP50 for >3 targets; (**c**) is EXT-67 for single targets; (**d**) is BMP67 for single targets. The *dashed lines* are the upper and lower bounds of the 95% prediction interval (PI95) for the FAHE model. The *dotted lines* (parts (**c**) and (**d**) only) are the upper and lower bounds of the 95% confidence interval (CI95) for the FAHE model. EXT = External contour. BMP = BrainMinusPTV

As shown in Fig. 5, the PI95 (dashed lines) of our FAHE model encompassed all but one individual data point across five independent publications [11, 12, 16, 18, 21]. The one point that fell above the PI95 was actually generated using a single-arc technique thus one would expect a somewhat higher irradiated volume, closer to SAHO. Furthermore, irradiated volumes V67 and BMP67 from two selected publications, were encompassed nearly entirely by the narrow CI95 (dotted lines) of the FAHE model, indicating FAHE was statistically equivalent to the cited publications [12, 16]. These results indicated that our framework can feasibly be applied across different publications and institutions as a means of comparison.

## Discussion

We have developed and tested a readily accessible framework for irradiated volume in HF-RT. The framework describes irradiated volume of the external contour (EXT) and Brain-Minus-PTV (BMP) for any given target volume and isodose level that any institution can implement for their technique and patient population. The purpose is not to seek out which technique is “better” or “worse”, but to simply facilitate a means for technique comparison.

The primary gain of the proposed framework is that despite the complex underlying physics behind it, irradiated volume can be reduced to a simple set of variables, which any institution can measure for their HF-RT patient population. Although we have limited the variables to target size, number, and shape for a given technique one could easily expand upon the model by adding other refining features, including MLC size, MLC margin around the target and number of arcs or isocenters. The clinical benefits of such a system include: (1) a framework through which institutions can perform treatment plan quality assurance, especially for complex situations such as HF-RT for which guidelines are lacking; (2) a framework through which treatment plans can be compared across institutions, which is often hampered by differing definitions of quality metrics. There are numerous published papers regarding the dosimetric benefit of one treatment technique over another, but the conclusions are often mixed, with some papers claiming little difference [12] and others citing larger differences [11]. The proposed framework may provide a means through which irradiated volume is parameterized and compared across institutions in a fair way. (3) a framework through which institutions can *improve* upon plan quality by reducing the variation in their own measured model over time. In our clinic, we have initiated using the proposed framework during the treatment plan review process by comparing irradiated volume parameters for upcoming plans against the model-predicted values. If a plan exceeds the prediction interval of the model, an investigation is undertaken to determine whether plan improvement can be made, and over time the spread of dosimetric data will diminish. Note that the prediction interval used in the present study was taken to be 95%, which was selected to cover an accurate range of dosimetric outcomes, at the cost of having wider intervals than using a less-predictive model such as with an 80% prediction interval.

As an example, a centre relatively new to HF-RT wishes to treat an inoperable and relatively large intact brain metastasis that has a volume (after PTV expansion) of 60cm^3^ with a fractionation scheme of 30Gy in five fractions, for which there is limited data on dose-volume constraints for normal tissue. The clinic is interested in the volume of tissue receiving >21Gy in five fractions, which for a 30Gy prescription is 70% of the prescribe dose, i.e. V70. From Fig. 1(c) and (d), if using a SAHO technique one may expect a range of V70 to be 119 cm^3^ ± 9 cm^3^ or 39 cm^3^ ± 11 cm^3^ for EXT and BMP respectively. If the clinic were willing to accept a hotter plan, with a target Dmax of up to 150% and planned with the FAHE technique, then the corresponding expected V70 would be lower than for SAHO (see Fig. 3d), in this case 83 cm^3^ ± 6 cm^3^ and 27 cm^3^ ± 8 cm^3^. If the target was irregularly shaped, or an additional target was also present, then the factors F_N_ or R could be appropriately applied. For any given isodose the same process can be applied, using the curves in Fig. 2 to extract the relevant parameters needed to asses irradiated volume. These ranges of irradiated volume serve as a guide of what may achievable: if a higher irradiated volume is achieved than the higher bound of the predicted range, then perhaps it may prompt a closer look at the complexity of the case or whether there can be improvements, whereas if a lower irradiated volume is achieved then that technique used is capable of achieving lower irradiated volumes, which may be a benefit.

An advantage of the present manuscript is that irradiated volume is described independently from prescription dose and PTV margin, and for both EXT and BMP, which facilitates comparison to published data. Certain treatment planning systems, such as Leksell GammaPlan, report irradiated volume to EXT whereas other planning systems can generate brain contours, and often subtract the PTV to define untreated brain, which we report as BMP. Irradiated BMP volumes are always less than EXT volumes (see Fig. 1), since the target and all dose spilling outside the brain contour are excluded from BMP. There is also more variation in the BMP curves: depending on the target location, more-or-less dose will spill beyond the brain into the skull and surrounding tissue. Our proposed framework also includes measuring EXT and BMP at multiple isodose levels such that any future isodose level can be interpolated, by presenting the fit parameters similarly as shown in Fig. 2.

Although we have demonstrated feasibility of our framework by comparing to internal and external data, there are certain limitations of the framework that need addressing. Firstly, our underlying clinical data is for single-arc HF-RT treatments. Although we re-planned 30 cases with a published FAHE technique, we do not use that approach clinically. Furthermore, our investigation of factors F_N_ and R may be limited to our treatment technique and patient population, which by virtue of being HF-RT, consisted of larger targets than those used for SRS. For example, our finding that having >3 targets resulted in higher (range: 1.4–2.9×) irradiated volumes than single-target cases may be due to increased dose interplay between targets resulting from the larger targets in our series (median and maximum volume = 14.1 and 84.6 cm^3^ respectively) than for typical SRS series. By contrast, the single metastasis model proposed by Bohoudi et al. consisted of target volumes in the range of 1 to 20 cm^3^ (median = 7 cm^3^) and was validated against multiple targets [16]. Naturally, there are many factors affecting the extent of irradiated volume, including penumbra, MLC margin around the target, beam modulation and number of arcs. However, despite the limitations, reasonable agreement was found comparing our FAHE model to data from other institutions demonstrating feasibility for inter-institutional comparisons [12, 20, 21]. This shows that at a minimum the specific factors we found can be used as a starting point going forward, and refinements can be easily incorporated. It would be interesting to present irradiated volume data acquired from other systems, such as the Gamma Knife or Cyberknife, in a similar format to that in the present manuscript.

It is important to emphasize that there are other factors to consider than irradiated volume when evaluating treatment planning techniques. For example, the conformity of the prescription isodose line to the target can vary between techniques. Another factor to consider is dose homogeneity. For HF-RT, our institution has consistently employed a 2 mm PTV margin, which has limited the comfort of the treating physicians to allow hotspots in the target in excess of 120–130%, as such steep gradients may cause normal-tissue necrosis within the margin itself. The dose limit of 130% is different from the situation of single-fraction SRS, in which the hotspot may easily extend up to 150–160%. Furthermore, the number of arcs and the degree of modulation can affect dose fall-off. The treating centre should determine what maximum allowed dose and degree of modulation it is comfortable with. Other important plan quality metrics for dose fall-off include the Paddick Gradient Index (GI), which is the ratio of volume receiving half of the prescribed dose to the volume receiving the prescribed dose. While GI is easy to measure, the expectation of the present work is that that normal tissue tolerances for HF-RT will ultimately be derived from clinical studies and reported in absolute volumes, much like the safety limit of V12 < 10 cm^3^ is used for SRS. One of the major strengths of the present work is there are no assumptions made as to what an “optimal” plan is for any given case, since treatment plan optimality depends on a multitude of factors. Rather than attempt optimality, our approach is heuristic in nature, and uses plans delivered to patients that the practising physicians (many of who have greater than 10 years experience in the field) deemed clinically acceptable.

Among other study limitations is the lack of clinical outcomes associated with the reported irradiated volume data. Although outcomes modeling is outside the scope of this work inclusion would clearly strengthen impact, and this work is ongoing. Another study limitation is that relatively few SAHO cases had more than 2 or 3 targets; having more multi-target cases would increase the accuracy of the determined F_N_. Although the framework can be extended to isodose lines <50%, 50% represents a clinically relevant lower limit [12]. Furthermore, in choosing to make the present manuscript readily comparable to other papers in the literature, we assumed that relative isodose lines are maintained irrespective of the absolute prescription dose whereas the planning process must incorporate machine constraints such as gantry speed.

## Conclusions

We have developed a framework for statistically describing irradiated volume in linac-based HF-RT. The model of irradiated volume we generated with clinical cases was validated against an independent internal dataset. We have also tested applying the framework to compare irradiated volume parameters against multiple published data. The framework is readily accessible to all institutions and independent of prescription dose or PTV margin.

## Abbreviations

BMP: Brain Minus PTV; sometimes referred to as “Normal” or untreated Brain in literature
CI95: 95% confidence interval
CTV: Clinical target volume
Dmax: Maximum dose
EXT: External contour (entire volume within external contour as defined on CT)
FAHE: Four-arc Heterogeneous
F_N_: Factor accounting for number (N) of targets
GTV: Gross tumor volume
Gy: Gray
HF-RT: Hypo-fractionated radiotherapy
MLC: Multi-leaf Collimator
MRI: Magnetic Resonance Imaging
P: Percentage of Prescription. Refers to specific isodose levels in percent
PI95: 95% prediction interval
PTV: Planning Target Volume
R: Factor accounting shape regularity
RI: Regularity index
RTOG: Radiation Therapy Oncology Group
SAHO: Single-arc Homogeneous
SRS: Stereotactic radiosurgery
TPS: Treatment planning system
TV: Target volume
V12: Volume receiving 12 Gy or greater
VMAT: Volumetric Modulated Arc Therapy
